# Neurosensory analysis of tooth sensitivity during at-home dental bleaching: a randomized clinical trial

**DOI:** 10.1590/1678-7757-2017-0284

**Published:** 2018-04-18

**Authors:** André Luiz Fraga Briso, Vanessa Rahal, Fernanda Almeida de Azevedo, Marjorie de Oliveira Gallinari, Rafael Simões Gonçalves, Paulo Henrique dos Santos, Luciano Tavares Angelo Cintra

**Affiliations:** 1 Univ. Estadual Paulista, Faculdade de Odontologia de Araçatuba, Departamento de Odontologia Restauradora, Araçatuba, São Paulo, Brazil; 2 Univ. Estadual Paulista, Faculdade de Odontologia de Araçatuba, Departamento de Materiais Odontológicos e Prótese, Araçatuba, São Paulo, Brazil

**Keywords:** Dental bleaching, Dentin sensitivity, Oxalates, Peroxides

## Abstract

**Objective:**

The objective of this study was to evaluate dental sensitivity using visual analogue scale, a Computerized Visual Analogue Scale (CoVAS) and a neurosensory analyzer (TSA II) during at-home bleaching with 10% carbamide peroxide, with and without potassium oxalate.

**Materials and Methods:**

Power Bleaching 10% containing potassium oxalate was used on one maxillary hemi-arch of the 25 volunteers, and Opalescence 10% was used on the opposite hemi-arch. Bleaching agents were used daily for 3 weeks. Analysis was performed before treatment, 24 hours later, 7, 14, and 21 days after the start of the treatment, and 7 days after its conclusion. The spontaneous tooth sensitivity was evaluated using the visual analogue scale and the sensitivity caused by a continuous 0°C stimulus was analyzed using CoVAS. The cold sensation threshold was also analyzed using the TSA II. The temperatures obtained were statistically analyzed using ANOVA and Tukey's test (α=5%).

**Results:**

The data obtained with the other methods were also analyzed. 24 hours, 7 and 14 days before the beginning of the treatment, over 20% of the teeth presented spontaneous sensitivity, the normal condition was restored after the end of the treatment. Regarding the cold sensation temperatures, both products sensitized the teeth (p<0.05) and no differences were detected between the products in each period (p>0.05). In addition, when they were compared using CoVAS, Power Bleaching caused the highest levels of sensitivity in all study periods, with the exception of the 14^th^ day of treatment.

**Conclusion:**

We concluded that the bleaching treatment sensitized the teeth and the product with potassium oxalate was not able to modulate tooth sensitivity.

## Introduction

At-home bleaching technique is recognized as a simple, biologically safe, and aesthetically effective therapy^4^ ^,^ ^10^ ^,^ ^17^. Studies established that reactive forms of oxygen promote oxidation of pigments, giving teeth a lighter appearance^10^. Upon penetrating the dental tissues, they quickly diffuse in the dental tissues reaching the chemosensitive ion channel (TRPA1), this activates the intradental nerves, causing discomfort^18^ ^,^ ^19^. Post-bleaching sensitivity was also related to the morphological changes that presumably alter the permeability, resulting in temporary sensitivity after the procedure^5^ ^,^ ^20^.

For this reason, products that repair these superficial changes are continually added in the composition of the bleaching agent^5^, blocking dentin tubules^2^ ^,^ ^5^ ^,^ ^6^, or acting in the transmission of nerve impulses, decreasing the ability of nerve fibers in the dental pulp to repolarize after an initial depolarization due to pain sensation^2^.

Some researchers verified that oxalates form crystals in the dental tissue and cause substantial changes in the dentinal fluid flow, reducing pain^11^. On the other hand, a systematic review by Cunha-Cruz, et al.^7^ (2011) concluded that available evidence suggests that oxalates are not effective in decreasing dentin hypersensitivity. They found a great variability across clinical trial leading to the need for standardization of pain stimuli and scales. Furthermore, potassium oxalate was recently added to an at-home bleaching product in an attempt to reduce sensitivity resultant from bleaching therapies.

To verify the clinical effects of the bleaching agents, researchers have employed methods based on experience reports^16^ and pain visual analogue scales^8^, considering the occurrence and intensity of spontaneous sensitivity. The results of these studies were important in the evolution of bleaching techniques and product development.

The TSA II-NeuroSensory Analyzer (Medoc; Ramat Yishai, Northern District, Israel) equipment has been used to quantify neurosensory responses from the larger and smaller fibers through provoked thermal stimuli, allowing the temperature that gives discomfort to the patient to be detected, this is an important information for studies on sensitivity^13^ ^,^ ^21^. This device has a central unit that generates and emits thermal stimuli through an intraoral probe. The patient holds a control device that stops the stimulus as soon as it is detected and the temperature at that moment is registered by the computer software. Thus, it is an interesting tool to analyze the discomfort felt by patients exposed to daily thermal stimuli. The accuracy of the method was highlighted by Gillam, et al.^11^ (2004), who revealed its efficiency when compared to other tests in a study of dental hypersensitivity.

Considering that dental sensitivity is the most common side effect after bleaching, a detailed study of its occurrence and intensity is important to establish comfortable therapies. This study aimed to evaluate the occurrence of spontaneous dental sensitivity and tooth sensitivity to cold produced through a thermal stimulus during and after whitening with carbamide peroxide, with or without oxalate.

The hypotheses tested were: (1) dental bleaching performed with carbamide peroxide with potassium oxalate added would reduce the intensity of spontaneous dental sensitivity, and (2) increase the dental sensitivity threshold under cold stimuli.

## Material and methods

### Experimental design

This clinical trial was approved by the Committee of Ethics in Research (00278/2011) and is registered in the Registro Brasileiro de Ensaios Clínicos (http://www.ensaiosclinicos.gov.br; register number: RBR- 4m94g4). The study was a randomized, split-mouth design, double-blinded and factorial clinical trial. The factors studied were: (1) two levels of bleaching treatment including 10% carbamide peroxide, and 10% carbamide peroxide containing potassium oxalate, and (2) six evaluating times including baseline, 24 h, 7, 14 and 21 days after the beginning of the treatment, as well as 7 days after the conclusion of the treatment.

Sample size was calculated for superiority trial, because we wanted to detect if one bleaching product would be more effective in reducing the intensity of the tooth sensitivity than the other. Furthermore, we considered the outcome intensity of tooth sensitivity for the sample size calculation and the variation used for this purpose was 5%.

We used a statistics calculator and it was considered the type-I error rate (α=5%) — the probability of finding a difference when a difference does not exist; type-II error rate (β=20%) — the probability of not detecting a difference when one actually exists, a 5% level of significance, which means that the chance of the finding being true is 95%; and a power of the test of 80%, which means that, if indeed there is any difference, the probability of detecting it will be 80%. Thus, the minimal sample size required was 10.

### Volunteer selection

#### Inclusion and exclusion criteria

The volunteers were healthy individuals between 18 and 25 years of age, who presented healthy oral soft tissues and vital maxillary lateral incisors with no carious or non-carious lesions and the baseline tooth color A3, according to the Vitapan Classical Shade Guide scale (Vita Zahnfabrik; Bad Säckingen, Baden-Württemberg, Germany).

Conversely, smokers, pregnant women, those who used braces, had received prior bleaching treatment, reported past adverse reaction to peroxides, were using opioids, were pacemaker patients, presented neurological, chronic or acute diseases, tooth sensitivity or treatment history of tooth sensitivity, dentin exposure, conoid teeth, direct or indirect restorations in the region of interest, tetracycline, injury, fluorosis or unknown staining, etiologies were excluded.

### Study groups

Thirty patients were recruited for the research, however, five of them did not meet the inclusion criteria. Thus, 25 volunteers underwent both bleaching treatments using products containing the same hydrogen peroxide concentration (10%). The treatment used in each hemi-arch was randomly determined by lottery. Thus, in one maxillary hemi-arch, Power Bleaching Gel 10% (BM4-Brazil Ltda. Materials and Instrumental; Palhoça, Santa Catarina, Brazil), which contains potassium oxalate, was used. The other maxillary hemi-arch was treated with Opalescence 10% (Ultradent Products Inc.; South Jordan, Utah, United States of America). Both bleaching products were dispensed in 3 ml-disposable opaque luer-lock syringes and randomly identified with codes A and B. The patients were told to use approximately 0.1 ml of the product in the area corresponding to the buccal surface of each tooth inside the tray. A researcher who did not participate in the clinical phase of the experiment performed the product and application drawings, so both volunteers and operators were not aware of the treatment they were undergoing in each hemi-arch.

### Bleaching treatments

Bleaching treatment was performed at the University Dentistry Clinic. Superior dental arch impressions were taken using alginate Jeltrate (Dentsply; Mildford, Delaware, United States of America) to obtain stone molds. The vestibular surface of the molds was coated with nail varnish, except for the last teeth of the arch. Over this mold a 1-mm-thick, 100% ethylene copolymer and vinyl acetate tray was fabricated for each volunteer in a vacuum-forming machine Plastvac P7 (Bio-art Equip. Odontol. Ltda.; São Carlos, São Paulo, Brazil). The trays were extended to the first molars, and cut at the marginal gingiva level. Each hemi-arch was randomly labeled with codes A and B to guide the volunteers to the correct use of the intended product in the proper quadrant. The volunteers were instructed to deposit one drop of the product on the bottom of the tray for each tooth to be bleached. The tray was then applied to the maxillary arch, and the excess product was removed with a toothbrush or gauze. The volunteers were informed of the dosage, and received a syringe of each product, which was applied daily for 4 h over the following three weeks. They were also instructed to use the trays during the afternoon, and to avoid brushing the teeth before that. All patients received oral hygiene kits to standardize the toothbrush and Oral B Pro-Saúde toothpaste (Procter & Gamble Manufactura; Naucalpan de Juárez, State of Mexico, Mexico) with fluoride in the composition. All treatments were monitored, since the volunteer returned to the clinic once a week.

### Spontaneous tooth sensitivity during and after treatment

**Figure 1:**
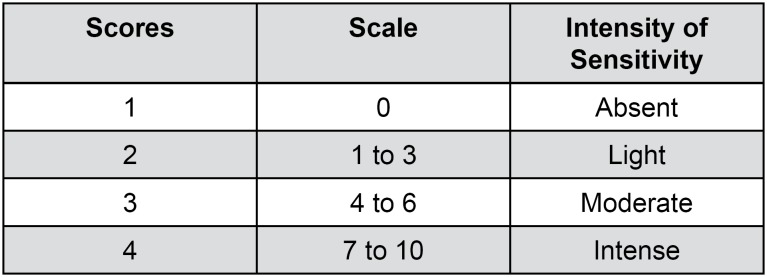
Scores related to the intensity of dental sensitivity

Spontaneous sensitivity was evaluated using a pain visual analogue scale two times a day, before and after the bleaching procedure. An intensity scale ranging from 0 to 10 (Figure 1) was established.

### Cold sensation on the teeth threshold analysis

**Figure 2:**
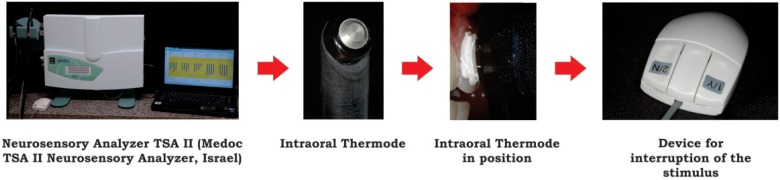
Scheme of the neurosensory analysis using the equipment TSA II

The analysis of cold sensation on the teeth was performed at the University Dentistry Clinic, in a calm and controlled environment at room temperature, at the six pre-determined evaluating times on the upper lateral incisors. For this procedure, a TSA-II Neurosensory Analyzer (Medoc; Ramat Yishai, Northern District, Israel) containing a delicate intraoral thermode was positioned in the flat region of the tooth surface (Figure 2). To standardize the dental region to be examined, a new 100% ethylene copolymer and vinyl acetate tray was manufactured in the same way described above. Each tray had circular perforations with a diameter similar to the active tip of the intraoral thermode. The perforations were done on the buccal tooth surface, on the flattest area of the teeth. Prior to analysis 0.02 mL of a thermally conductive paste containing a silver oxide base IPT - Implastec Thermal Grease (Implastec Ltda Electrochemical; Votorantim, São Paulo, Brazil) at room temperature were applied in this are to optimize thermal conduction.

The test was performed in a dental office between 8:00 a.m. to 10:00 a.m., in a quiet environment with a constant temperature of 26°C. To verify the cold sensation threshold (CST), the TSA II analyzer was configured in the “Limits” function, where three tests with descending temperatures (from 36°C to 0°C) were performed at each time of evaluation. Thus, three temperatures were recorded, and the average temperature was used as the result. Each test initiated at 36°C, with a temperature cooling speed rate of 1°C/s. When the thermal stimulus was detected, it was immediately interrupted by the patient. After that happened the temperature detection was recorded, and from that point forward, the temperature was gradually increased until the comfort temperature (36°C) was restored. This temperature remained constant for 20 s, and another analysis took place. All analyses were performed three times.

### Computerized Visual Analogue Scale (CoVAS)

To evaluate dental sensitivity during the bleaching treatment in the six pre-set study periods, the Computerized Visual Analogue Scale (CoVAS) (Medoc; Ramat Yishai, Northern District, Israel), which is part of the TSA-II Neurosensory Analyzer hardware accessories package, was used in real time, after the dental cold sensation threshold analysis. A constant thermal stimulus (0°C) was applied to the upper lateral incisors for 15 seconds. During this period, the volunteer recorded the intensity of discomfort experienced on a scale of 0 to 100, using a manualcontrolled potentiometer. The responses obtained were synchronized, to compare the dental sensitivity generated by the different treatments in individual volunteers.

### Clinical analysis of gingival condition

The clinical analyses of periodontal condition were performed once a week during all treatments. For this purpose, a dental mirror and artificial light were used. During the procedure, the presence or absence of bleaching-related gingival irritation around the teeth that underwent the bleaching procedure was evaluated, and the following scores were assigned: (0) healthy gingiva or (1) gingiva altered by erythema, swelling, bleeding, or a shiny surface.

### Statistical analysis

The spontaneous sensitivity was recorded before and after the bleaching treatment and ranked, according to previously established scores to determine the results. The average temperature (°C) calculated after the three descending temperature tests made at each moment of evaluation during the cold sensation threshold analysis for both bleaching materials was submitted to analysis of variance (ANOVA) and Tukey test (level of significance of 0.05).

The intensity of discomfort experienced on a scale of 0 to 100 obtained using Computerized Visual Analog Scale (CoVAS) and clinical analysis of gingival condition were submitted to descriptive analysis.

## Results

**Figure 3:**
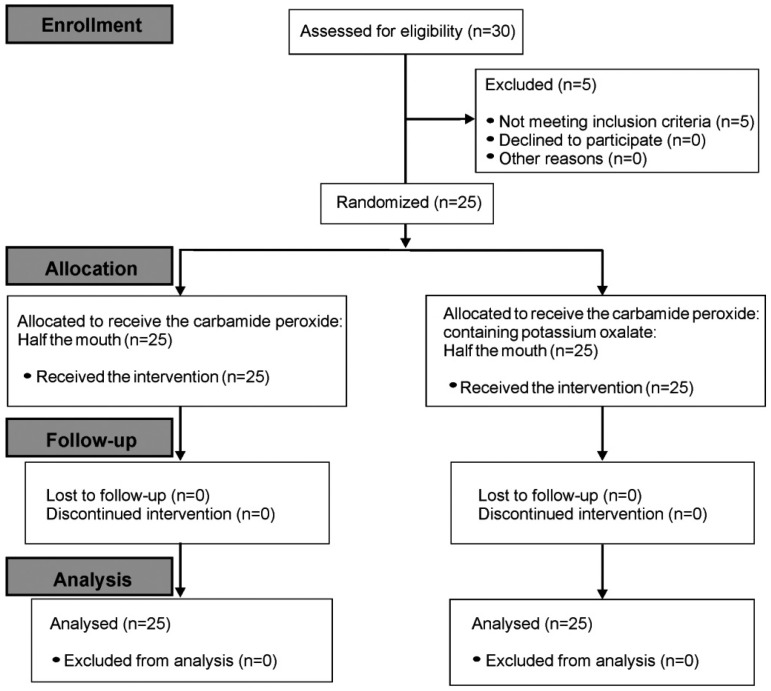
Flow-chart diagram of the volunteers selection detaching the enrollment, allocation, follow-up and analysis during the study (CONSORT Statement)

Thirty patients were recruited for the study, but five did not meet the pre-determined inclusion criteria. Thus, 25 volunteers received the proposed treatment and were included in the analysis performed. No losses were verified for the study groups (Figure 3).

### Spontaneous tooth sensitivity

The spontaneous sensitivity analysis showed that in most cases, the volunteers presented no discomfort during treatment. Thus, 6 patients (24%) had tooth sensitivity 24 hours before using the product Power Bleaching 10% (T2), 10 patients (40%) presented the discomfort 7 days after treatment initiation (T3) and after 14 days (T4), 8 patients (32%) reported sensitivity. On the other hand, 7 (28%), 6 (24%) and 7 (28%) patients in T2, T3 and T4, respectively, presented the discomfort using the product Opalescence 10%

**Figure 4:**
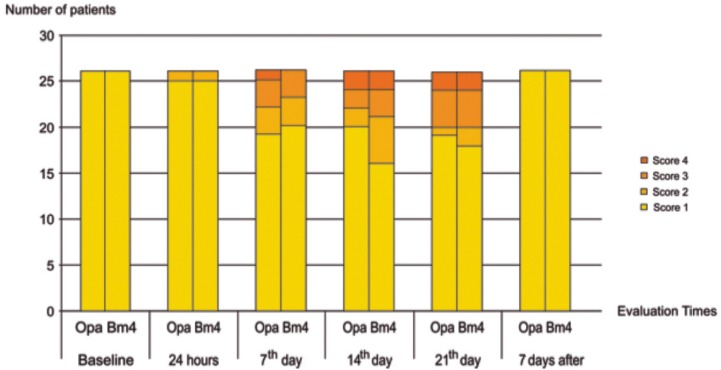
Number of patients that reported spontaneous sensitivity during the bleaching treatment according to the study period and bleaching product

Patients using Power Bleaching 10% mainly reported sensitivity 7 days after starting the treatment, mostly at a low-level, however, scores similar to the baseline were restored after the end of the treatment, with no volunteers presenting tooth sensitivity in both groups (T5; Figure 4).

### Dental cold sensation threshold

We applied the Tukey test to compare the performance of both bleaching materials. We verified that both products sensitized the teeth (p<0.05) and no significant differences were detected between the bleaching products in the six study periods (p>0.05). Thus, emphasizing that the higher the temperature, the more sensitive the tooth was, is important (Table 1).

**Table 1:** Average cold sensation temperatures (±standard deviation) obtained in the study periods according to the bleaching product

Time / Products	Baseline	24 hours	7^th^ day	14^th^ day	21^st^ day	7 days after
Opalescence	11.31(3.39)^A^ *	16.62(3.63)^B^	15.77(3.77)^B^	16.56(4.00)^B^	15.92(3.85)^B^	14.55(3.86)^B^
Power Bleaching	11.44(3.53)^A^	16.96(3.21)^B^	16.69(3.56)^B^	16.73(4.13)^B^	16.07(3.94)^B^	15.25(3.83)^B^

* Means followed by different letters, capital letters in the rows are statistically different (p<0.05)

### Computerized Visual Analogue Scale (CoVAS)

Dental sensitivity in both study groups was observed on all study periods when a continuous thermal stimulus (0°C) was applied (CoVAS test).

**Figure 5:**
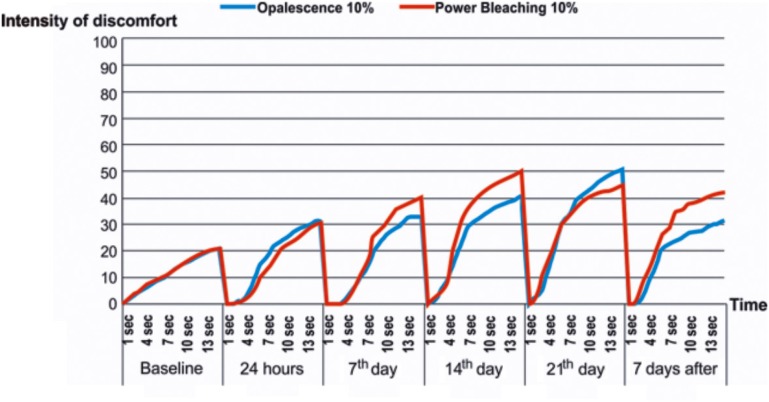
Computerized Visual Analog Scale (CoVAS) scale obtained using a 0°C constant temperature for 15 seconds, according to the study periods and bleaching products

Figure 5 shows that the bleaching treatments produced curves with peaks that show the moment of the highest intensity of sensitivity that the patient experienced. In this case, Power Bleaching caused the highest intensity of dental sensitivity in all study periods (T1, T2, T3, and T5), but T4.

### Gingival condition

**Figure 6:**
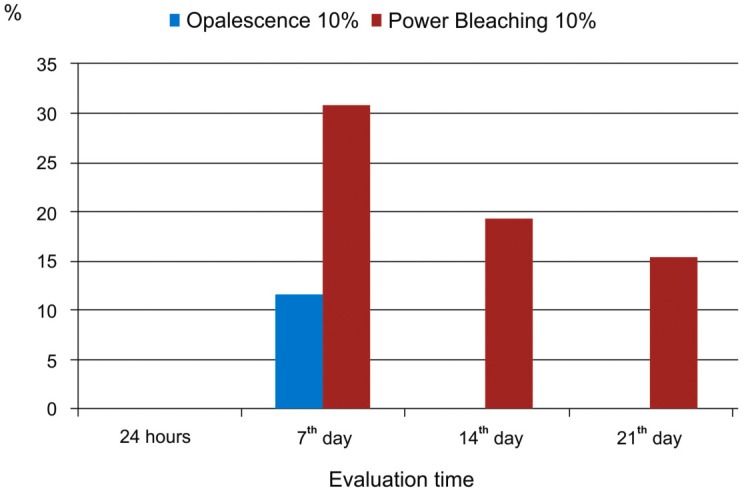
Volunteers (%) presenting gingival irritation during the bleaching treatment, according to the study periods and bleaching products

Generally, the results indicated that the product containing potassium oxalate caused more gingival irritation than the Opalesence 10% (Figure 6).

During the bleaching treatment, approximately 30% of the volunteers who used the Power Bleaching product showed gingival irritation in T2. This percentage decreased in other study periods, and was approximately 20-15% of volunteers in T3 and T4, respectively. On the other hand, volunteers who used the Opalescence 10% presented gingival irritation only in T2 (12%).

Lastly, we note that although color alteration was not analyzed in our study, both bleaching products used for bleaching approache promoted highly satisfactory outcomes, with lighter and aesthetically improved teeth.

## Discussion

Although bleaching treatments are considered a conservative and effective option for treating chromatic dental alterations, at-home bleaching products based on 10% carbamide peroxide may cause transient discomfort and dental sensitivity^9^ ^,^ ^16^.

Some *in vitro* studies report morphological changes in enamel during the bleaching treatment that are related to the occurrence of dental sensitivity^22^ ^,^ ^25^. However, these studies do not reproduce the complex process on tooth demineralization and remineralization^14^. The continuous presence of saliva associated with the correct dosage is believed to guarantee the maintenance of the dental enamel surface characteristics, little influenced by dental sensitivity.

Other authors have reported that the penetration of hydrogen peroxide from the bleaching agents promote the activation of the ion channel TRPA1, present in some of the intradental nerves, which is implicated in the mediation of pain induced by cold^18^ ^,^ ^19^.

Due to frequent reports of sensitivity during bleaching, desensitizing agents were incorporated into the composition of the bleaching products^5^. Given this context, potassium oxalate was added to the bleaching product Power Bleaching 10%, aiming to decrease the collateral effect through the formation of precipitates in the tooth structure, reducing permeability and fluid movement within the dentinal tubules^24^. In addition, some authors stated that oxalate salts also block neural transmission at pulp level^23^.

Spontaneous sensitivity was observed during all treatment periods of this study, mostly at a low-to-moderate level, but it was more frequently reported in the group treated with the Power Bleaching 10% product. This result allowed us to reject the first hypothesis of this study. These data corroborate with the literature that reports that a sizable proportion of the patients experienced sensitivity during and after bleaching treatment^1^ ^,^ ^3^.

On the other hand, when the continuous thermal stimulus was applied (CoVAS test), except in T4, higher levels of dental sensitivity in teeth treated with Power Bleaching 10% were identified, with sensitivity persisting after the end of the treatment.

The application of the quantitative sensory test showed that, regardless of the bleaching product used, the teeth became more sensitive and temperature changes were more rapidly detected after the first day of treatment. This result led to the rejection of the second hypothesis of this study.

Noting that the devices used provide reproducible and reliable tests, especially when conducted by a single operator and associated to the collaboration of the patient is important. Additionally, these modern and efficient equipment allow the quantification of the painful symptoms, through neurosensory analysis, the Quantitative Sensory Testing (QST)^13^. The major advantage of this technology is allowing the evaluation of thick and thin myelinated and unmyelinated fibers, which is important since different types of fiber penetrate the dental apical foramen^19^ ^,^ ^21^.

Pain visual analogue scale, CoVAS and TSA II were used because they are easily applied and well tolerated by the patients. These characteristics are important since a time-consuming analysis causes stress and data inaccuracy.

Furthermore, sensitivity changes during specific situations, such as systemic disease and ageing^15^. Thus, sample standardization regarding health condition, gender and age should be established since those factors may influence the results.

The results of this study revealed that the bleaching product containing oxalate showed little to no effect on the control and occurrence of dental sensitivity and did not exceed the performance of bleaching agents without desensitizers in any of the tests in this study, which demonstrably showed a lower performance than various products containing these agents^1^. Given this context, we emphasize the systematic review performed by Cunha-Cruz, et al.^7^ (2011) that concluded that available evidence suggests that oxalates are not effective to decrease sensitivity.

The considerable occurrence of gingival tissue irritation in the hemi-arch treated with the bleaching agents that contained oxalate should also be emphasized. Consequently, we can infer that the presence of this salt may be a primary cause of the irritation. Another hypothesis to consider is the pH difference between the bleaching products used. Although the two products had pH values close to neutral, the bleaching agent that contained oxalate was slightly more acidic. However, although there are reports of gingiva sensitivity during the bleaching treatment, there are no reports of the bleaching treatment causing severe harm to the gingiva^16^.

Nevertheless, some reports still report the efficiency of oxalate salts in controlling sensitivity when applied directly on the dental tissues^11^ ^,^ ^24^, stimulating new comparative studies, relating the work of different concentrations of oxalate with its cytotoxicity, collateral effects, and effectiveness^12^ ^,^ ^23^.

## Conclusions

The results obtained in this study allow us to affirm that dental bleaching performed with carbamide peroxide with potassium oxalate in the composition did not modulate the occurrence and intensity of spontaneous sensitivity, nor reduced the dental sensitivity threshold when a thermal stimulus was used.
