# ERRATUM: Neurosensory analysis of tooth sensitivity during at-home dental bleaching: a randomized clinical trial

**DOI:** 10.1590/1678-7757-2018-er001

**Published:** 2018-06-25

Due to a publishing error the article “Neurosensory analysis of tooth sensitivity during at-home dental bleaching: a randomized clinical trial”, published at Journal of Applied Oral Science 2018;26:e20170284 was printed with the following error:

**Page 1/8**

Authors:

Where you read:

**André Luiz Fraga BRISO** ^**1**^ **, Vanessa RAHAL** ^**1**^ **, Fernanda Almeida de AZEVEDO** ^**1**^ **, Marjorie de Oliveira GALLINARI** ^**1**^ **, Rafael Simões GONÇALVES** ^**1**^ **, Paulo Henrique dos SANTOS** ^**2**^ **, Luciano Tavares Angelo CINTRA** ^**1**^

1- Univ. Estadual Paulista, Araçatuba Dental School, Department of Restorative Dentistry, Araçatuba, São Paulo, Brazil.

2- Univ. Estadual Paulista, Araçatuba Dental School, Department of Dental Materials and Prosthodontics, Araçatuba, São Paulo, Brazil.

Should be:

**André Luiz Fraga BRISO** ^**1**^ **, Vanessa RAHAL** ^**1**^ **, Fernanda Almeida de AZEVEDO** ^**1**^ **, Marjorie de Oliveira GALLINARI** ^**1**^ **, Rafael Simões GONÇALVES** ^**1**^ **,** **Sandra Meira Borghi Frascino** ^**1**^ **, Paulo Henrique dos SANTOS** ^**2**^ **, Luciano Tavares Angelo CINTRA** ^**1**^

1- Univ. Estadual Paulista, Araçatuba Dental School, Department of Restorative Dentistry, Araçatuba, São Paulo, Brazil.

2- Univ. Estadual Paulista, Araçatuba Dental School, Department of Dental Materials and Prosthodontics, Araçatuba, São Paulo, Brazil.

